# Enteroflow: Automated Pipeline for In Silico Characterization of *Enterococcus faecium*/*faecalis* Isolates from Short Reads

**DOI:** 10.3390/ijms26199441

**Published:** 2025-09-26

**Authors:** Daniele Smedile, Elena L. Diaconu, Matteo Grelloni, Barbara Middei, Virginia Carfora, Antonio Battisti, Patricia Alba, Alessia Franco

**Affiliations:** National Reference Laboratory for Antimicrobial Resistance, Department of General Diagnostics, Istituto Zooprofilattico Sperimentale del Lazio e Della Toscana “M. Aleandri”, 00178 Rome, Italy; daniele.smedile@izslt.it (D.S.); elena.diaconu@izslt.it (E.L.D.); matteo.grelloni15@gmail.com (M.G.); barbara.middei-esterno@izslt.it (B.M.); virginia.carfora@izslt.it (V.C.); antonio.battisti@izslt.it (A.B.); alessia.franco@izslt.it (A.F.)

**Keywords:** antimicrobial resistance (AMR), nNextflow pipeline, *Enterococcus* spp., illumina sequencing

## Abstract

Antimicrobial resistance (AMR) is a critical global health challenge that affects both human and animal populations. In accordance with the One Health paradigm, AMR has been monitored in Italy since 2014 in major zoonotic pathogens and opportunistic commensal bacteria from animal productions, in the frame of the EU Harmonized Monitoring Program for AMR (according to EU Decision 2013/652, repealed by EU Decision 2020/1729), conducted by the Italian National Reference Center (CRN-AR) and National Reference Laboratory (NRL-AR) for antimicrobial resistance at the “Istituto Zooprofilattico Sperimentale del Lazio e della Toscana (IZSLT)” (on behalf of the Italian Ministry of Health). Among all monitored bacterial species, the commensal *Enterococcus* (*E.*) *faecium* and *E. faecalis* have emerged as opportunistic human pathogens with increasing AMR profiles. To address this challenge, the CRN-AR and NRL-AR have developed a custom bioinformatic pipeline, named Enteroflow, which enables the efficient analysis of high-throughput sequencing (HTS) data for the genomic characterization of *E. faecium*/*faecalis* isolates. A pivotal feature in this tool is the integration of Nextflow’s workflow manager and Domain Specific Language (DSL), ensuring the reproducibility and scalability of genomic analyses while allowing the monitoring of processes and computational performances. The list of tools included in the workflow spans from short read assemblers to genomic characterization tools for AMR and virulence gene detection and plasmid replicon typing, with results also being combined in structured and usable reports. These developments represent a major step forward in supporting the surveillance efforts and mitigation strategies for AMR in zoonotic and commensal bacteria.

## 1. Introduction

AMR (antimicrobial resistance) represents one of the main public health concerns of the 21st century, affecting both animal and human sectors. Following a One Health perspective, AMR zoonotic agents have become a public health issue, given their ability to arise and spread between humans, animals, and wildlife. Therefore, AMR has been monitored in Italy since 2014 in major zoonotic pathogens (*Salmonella* spp., *Campylobacter jejuni*/*coli*) and indicator commensal bacteria (*Escherichia coli* and *Enterococcus faecium*/*faecalis*) originating from animal productions, in the frame of the EU Harmonized Monitoring Program for AMR (according to EU Decision 2013/652 [1], repealed by EU Decision 2020/1729/EU [2]) conducted by the Italian National Reference Center (CRN-AR) and National Reference Laboratory (NRL-AR) for AMR, based on the “Istituto Zooprofilattico Sperimentale del Lazio e della Toscana (IZSLT)” in cooperation with the Italian Ministry of Health. *E. faecium* and *faecalis* are two common opportunistic pathogens with a growing incidence of AMR, *E. faecium* included among the ESKAPE pathogens by the World Health Organization (WHO) [3], and both are targeted as indicator commensals to be voluntarily monitored for AMR by EU Member States, according to the Commission Implementing Decision (EU) 2020/1729 [2]. These are ubiquitous Gram-positive bacteria from the *Enterococcus* genus that inhabit the gastrointestinal tracts of humans and animals. While traditionally regarded as commensal organisms, certain species—most notably *E. faecalis* and *E. faecium*—have drawn attention as significant zoonotic pathogens, contributing to infections in immunocompromised individuals and hospital settings [4]. In animals, enterococci function primarily as opportunistic microbiota, yet they can act as indicators of gut health and environmental exposure. Their capacity to acquire and disseminate AMR genes has heightened interest in their surveillance, representing sentinel organisms for assessing antimicrobial selective pressure, particularly in response to the use of antibiotics in veterinary and agricultural contexts [5]. Their resilience and genomic adaptability render them valuable indicators of anthropogenic impact on microbial ecosystems, primarily mediated by horizontal gene transfer (HGT) mechanisms of mobile genetic elements (MGEs), which promote the transferability of resistance determinants [4]. *E. faecium*/*faecalis* show intrinsic resistance to certain antibiotic classes and molecules such as cephalosporins, aminoglycosides (low-level resistance), clindamycin (*E. faecalis*), and sulfonamides, combined with their ability to develop resistance to high-level aminoglycosides, oxazolidinones, and glycopeptides, with the vancomycin-resistant enterococci (VRE) posing the greatest threat for morbidity, mortality, and healthcare costs in the human sector. Enterococci may carry and horizontally transmit between different lineages and populations a significant number of said genes, which confer antibiotic resistance and pathogenic capabilities through mobile genetic elements (MGEs), like plasmids or transposons. Therefore, *Enterococcus*’s genomic dynamics make it a very adaptable emerging opportunistic pathogen as its capacity to acquire, transmit, and lose such genetic features enhances the likelihood of the development of new infectious variants and resistance combinations [6].

In the frame of the AMR surveillance activities on food-producing animals (EU harmonized monitoring) carried out since 2014, the CRN-AR/NRL-AR has manifested the need to analyze increasingly vast amounts of complex sequencing data produced by high-throughput sequencing (HTS) instruments, to characterize and identify the genetic basis of AMR and other significant genetic features of zoonotic and commensal bacteria, including *Enterococcus* species.

Hence, this prompted the development of a custom bioinformatic pipeline named Enteroflow, which could optimize the various phases and coordinate the numerous applications involved in the analysis of large amounts of bacterial genomic data in the most time-efficient way possible. For this purpose, a scientific workflow management system named Nextflow (v. 24.10.5 build 5935) [7], conceived for creating scalable, portable, and reproducible pipelines, was the most logical choice. It is based on the dataflow programming model and uses its homonymous Domain Specific Language (DSL) based on Apache Groovy. Nextflow’s workflow manager was adopted because of its ability to manage software dependencies and to make the workflow reproducible and scalable as its DSL offers a high-level parallel computing environment, aimed at organizing the interactions among processes. In addition, the user can monitor performances and dynamically manage the workload by consulting metrics and existing files. Nextflow [7] natively allows the integration of various scripting languages, both for file manipulation (bash, python) and integrated data analysis (R and Markdown), with the production of interactive .html reports providing immediate feedback on the pipeline’s status.

## 2. Results

### 2.1. Pipeline: Enteroflow

**Figure 1:**
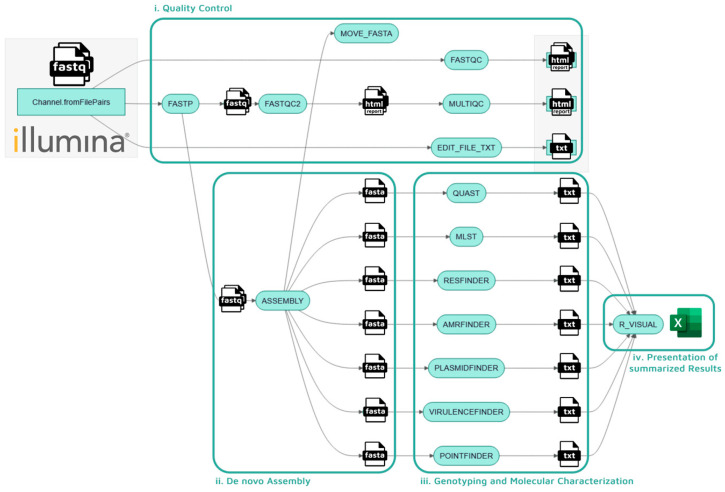
Scheme of Enteroflow’s workflow from input (left) to output (right), highlighting the 4 main phases of the pipeline, numbered as i, ii, iii, and iv. Icons are only representative of file formats for each tool’s input/output.

The final version of the pipeline, named Enteroflow, is divided into four main sections, which are described extensively in the Material and Methods chapter: (i) quality control of input raw reads, (ii) de novo assembly, (iii) genotyping and molecular characterization, and (iv) reporting of summarized results (Figure 1).

### 2.2. Performance Results

The pipeline was officially tested on two different sample sets: the first one was intended for quality testing to evaluate the pipeline’s fitness and comprised five short-read, paired-end sequenced isolates belonging to the PRJNA433676 project; the second one was used to test computational performance and hardware requirements with a total of 761 *Enterococcus* isolates from the 2023 Harmonized Monitoring Program on AMR conducted in Italy.

#### 2.2.1. Quality Testing Results

Five short, paired end reads, of publicly available *E. faecium* isolates were downloaded from NCBI onto the IZSLT’s server and submitted to the pipeline. For this analysis, 14 threads and 64 GB of RAM were used. The total elapsed time was 30 min and 18 s.

**Figure 2:**
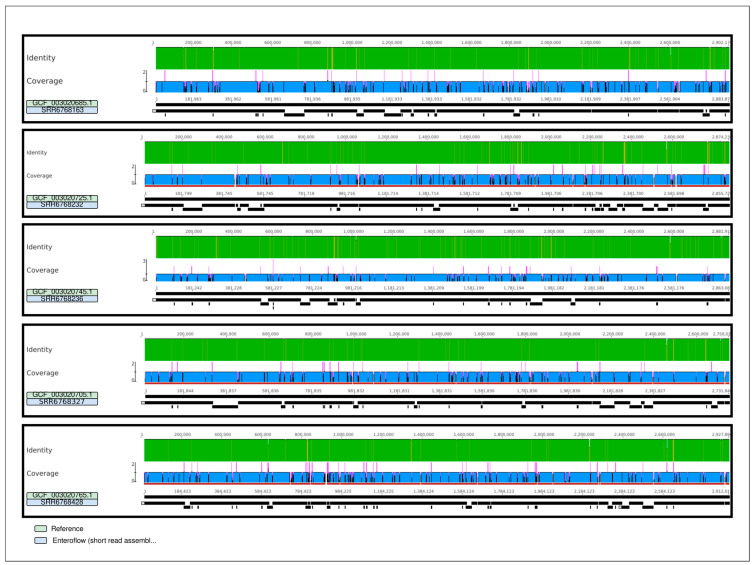
Alignment of 5 assemblies produced by Enteroflow with short reads retrieved from the PRJNA433676 project versus the corresponding reference sequences produced via PacBio sequencing from the same project. Samples are divided in boxes, with Enteroflow’s assembled genome at the bottom and the related reference right above it. Coverage follows the scale shown on the y axis(left), with values represented both in height and colour (darker is lower, lighter is higher). Identity with the reference genome is shown on a scale from 0 to 1, with green for perfect matches (100% identity) and yellow for lower values.

Final assemblies obtained from raw reads via Enteroflow were mapped against the publicly available complete genomes of the same isolates, generated by PacBio’s long read sequencing technology. Results show that contigs produced by Enteroflow cover the genome of each *E. faecium* isolate, with a deviation of under 6% in size (coverage range: 93.7–95.1%) in all five cases (Figure 2 and Table 1).

#### 2.2.2. Computational Performance Testing

**Figure 3:**
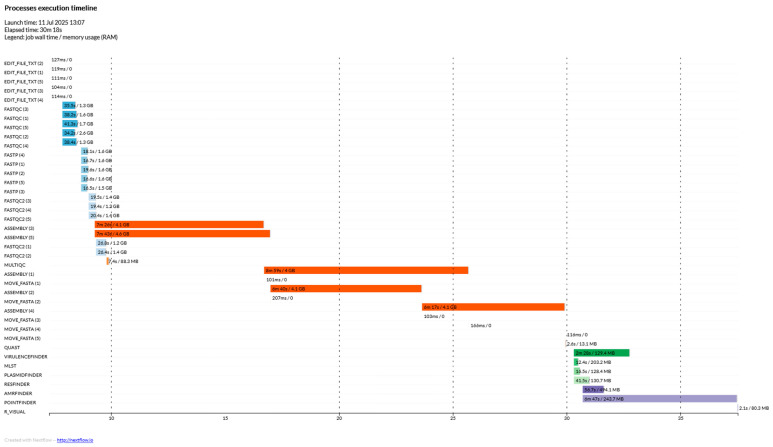
Process execution timeline of the quality testing run, involving 5 *Enterococcus* spp. isolates from the PRJNA433676 project. The y-axis shows the different processes in chronological order (top to bottom) while the x-axis represents the elapsed time in minutes.

**Figure 4:**
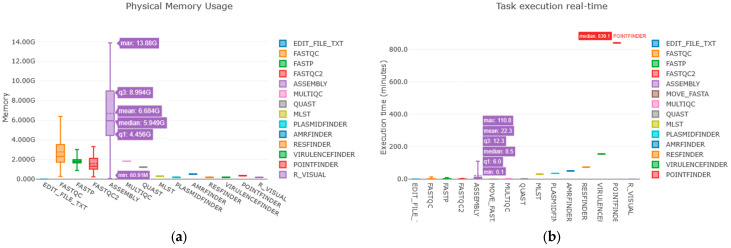
Performance testing results following the analysis of 761 genomes of *Enterococcus* spp. with Enteroflow. (**a**) Physical memory usage candlestick chart: The *y*-axis represents the amount of memory used (in Gigabytes) by all the tools represented on the *x*-axis. (**b**) Task execution time candlestick chart: The *y*-axis shows the time of execution of each process expressed in minutes.

The pipeline’s performance testing was carried out by simultaneously analyzing 761 *Enterococcus* spp. isolates in a run with 30 threads and 256 GB of RAM available. The elapsed time added up to 3 days, 13 h, 44 min, and 15 s, which corresponded to 209.4 CPU-hours thanks to the intrinsic parallelization capabilities of Nextflow’s DSL. Peak RAM usage reached 13.9 GB, underlining the possibility of running Enteroflow on less computationally capable devices (Figure 3 and Figure 4).

The resulting assemblies and downstream analysis have proven to be coherent with previous phenotypic analyses of the same isolates, regarding both species’ identification and resistance characteristics (Supplementary Table S1).

### 2.3. Final Output

Output results from all processes are stored inside the data folder created inside the user-selected running directory (“TEST” environment variable). Information regarding the quality controls and filtering of raw reads are stored inside the data/fastp and data/multiqc folders. Files generated by the genotyping and molecular characterization phase (see Materials and Methods), in .txt format, are saved in the data/annotation subdirectory and summarized in a single comprehensive excel file by a custom R script. These include information on the quality of assembly, Multi-locus Sequence Typing (MLST), accessory resistance genes identified using ResFinder and AMRFinder databases, specific point mutations involved in AMR identified using PointFinder, plasmid replicons from PlasmidFinder, and virulence genes identified using the VFDB. The excel workbook is organized in six different sheets named after the corresponding tools, containing results from all analyzed isolates together. This approach is intended to facilitate accessibility and interpretability of results, even for users who are not familiar with command-line interfaces.

### 2.4. Benchmarking Test

Our pipeline has been compared with other well-known pipelines: Nullarbor (v. 1.41) and Bactopia (v. 3.1.0). Table 2 hereafter shows the resulting execution time, maximum ram usage, and type of output from the different pipelines.

Enteroflow, besides the single results from the included tools, produces a compiled excel file containing the MLST, the resistance genes detected with two different databases (ResFinder and AMRFinder), the species-specific point mutations (*Enterococcus faecalis* or *faecium*), the plasmid replicons, the virulence genes, and basic statistics about the quality of the assembly (Supplementary Table S1). Optionally, taxonomic classifications via kraken2 and the production of a similarity tree are available.

Bactopia, which is written in Nextflow’s DSL just like Enteroflow, produces different tsv files resulting from the various included tools. These, among others, regard the detection of MLST and antimicrobial resistance genes via AMRFinder, assembly results, quality control, and whole genome annotation via Prokka (Supplementary Table S2).

Nullarbor, on the other hand, does not rely on Nextflow’s environment and is oriented towards a more classical script-like structure, which implies bigger differences with Enteroflow. One of the main differences from the other pipelines lies in the requirement of a reference genome, which is essential to produce results regarding the SNPs phylogeny and alignment but in the meantime limits the use to samples which belong to the same species and are very closely related at the genomic level. Other results include csv files presenting results from MLST, detected resistance and virulence genes with related data, quality of assembly, and annotation of the produced genomes (Supplementary Table S3).

## 3. Discussion

Recently, in Italy, healthcare settings have observed an increase in infections caused by *E. faecium* [8] along with a rising percentage of VRE (https://www.epicentro.iss.it/antibiotico-resistenza/ar-iss-rapporto-enterococcus-faecium (accessed on 23 July 2025)) in the human sector. Moreover, a strong correlation between the presence of VRE *E. faecium* as commensals in feces and bloodstream infections caused by these vancomycin-resistant microorganisms has been observed in healthcare settings [9]. As per the animal sector, the Italian CRN/NRL-AR adheres, since 2023, to the voluntary monitoring of the commensal indicator species of *E. faecalis* and *E. faecium* from animal productions (Commission Implementing Decision (EU) 2020/1729 [2]), aiming at actively monitor AMR in *Enterococcus faecium*/*faecalis* species. This effort results each year in a large amount of *Enterococcus* spp. isolates, which are tested for their antimicrobial susceptibility according to current legislation, then WG-sequenced and subjected to bioinformatics analysis. The introduction and implementation of Enteroflow have significantly optimized the dry-lab component of the WGS workflow implemented at the CRN NRL-AR, in terms of the efficiency of the analysis/interpretation of results from large amounts of genomic raw data.

During recent years, different pipelines have been built to optimize the assembly and AMR-related annotation of prokaryotes [10,11,12,13,14], although, to the best of our knowledge, none of them were intended to study the molecular basis of AMR in *Enterococcus* spp., including the identification of point mutations in chromosomic genes conferring resistance to specific antimicrobial classes as aminopenicillins. Enteroflow was designed to reproduce a harmonized bioinformatic analysis, starting from quality checks on raw reads and ending with a user-friendly output. Results are summarized in a single excel file which includes information on quality of assembly, MLST, accessory resistance genes, point mutations involved in AMR, plasmid replicons, and virulence genes. The production of a comprehensive excel file containing all the data was intended to allow any microbiologist or clinician with basic IT skills to perform a manual evaluation of the results, relieving the bioinformatician from this task. Furthermore, all assembled genomes are available for the user in .fasta format together with pre/post-filtering reports on read quality.

The installation process is under constant development aiming at a seamless and intuitive experience for the end user. Although using Enteroflow requires a minimum knowledge of bash’s command line and of other tools such as Nextflow [7] or Conda [15], we believe the process has been simplified to an extent where any bioinformatician will be able to handle it with ease.

Other than a GNU operating system and a Linux kernel, the only other requirements regarding the installation of software on which the pipeline relies are as follows:

First and foremost is Nextflow [7] itself, which obviously must be installed on the machine we would like to run the pipeline on. The second paramount software is Conda’s package management system [15], by which the pipeline is able to create separate environments for the various tools in the workflow, also handling all required packages. Unfortunately, the most recent supported version is Conda 25.1.1, as some deprecations in the latest releases conflict with the pipeline’s commands. Our future intention is to provide a new released version for Enteroflow (0.1), which will be able to run with the latest Conda versions. Also Blast (v2.17.0) [16] and KMA (v1.5.1) [17] must be installed separately by the user before the pipeline may run, as many of the tools rely on these aligners for genome assembly and annotation.

Should the user choose to include the available taxonomical classification feature via Kraken2 (v2.1.6) [18] and MashTree (v1.4.6) [19], this first tool will require a separate installation process. These options have been made discretionary due to the size of Kraken’s database (approximately 90 GB), which may represent an impediment for smaller IT systems.

Tests performed for the validation of the pipeline have proven its efficiency with a peak of maximum RAM use around 14 GB and a total time of 3 and a half days to process 761 isolates, representing an average number of samples produced throughout one year of monitoring.

Unlike many other published pipelines, Enteroflow does not annotate the resulting assemblies with tools like Prokka or Bakta [10,11,12,13,14], significantly reducing the complexity of the output and making it accessible to any professional with little to no IT knowledge required. Inter-sample genomic comparison has been relegated to the simple MLST (typing scheme from Homan [20] and Ruiz-Garbajosa [21]), and taxonomical classification steps have been rendered optional with the intent of keeping the workflow as nimble as possible while still providing the user with necessary information.

Some major differences, among others, have stood out during benchmarking tests, particularly regarding the structure and the type of results generated by these pipelines. Nullarbor (v1.41), for example, is the only one requiring a reference genome for the complete analysis of the isolates, which allows for SNPs detection but may be challenging in non-outbreak-related contexts like the monitoring of *E. faecalis* and *E. faecium* in food-producing animals. Moreover, this is the only pipeline which does not produce a summary of the overall assembly quality, which we added in Enteroflow to ensure reliability of results.

Bactopia (v3.1.0) on the other hand, has a similar structured output when compared with Enteroflow, adding whole genome sequencing via Prokka (v1.14.5) or Bakta (v1.11.4) while still lacking the detection of AMR-related point mutations for *Enterococcus* spp. Unfortunately, while preparing Bactopia to run all 10 samples consecutively, we encountered errors in the accessory tools required for the creation of the sample list and could not solve the problem manually either. This forced us to run the pipeline in 10 different single-isolate runs, resulting in further delays between runs and the need for continuous intervention by the user. Results from these single runs have been merged manually and duration times have been summed, with RAM usage peak being a mean of the ten observed values.

Regarding AMR gene detection, all three pipelines produce results regarding resistance genes and related data. In detail however, Nullarbor utilizes the ResFinder database by default, Bactopia relies on the AMRFinder database, while Enteroflow uses them both for reliability and comparison of results. Furthermore, Enteroflow’s specificity allows it to detect point mutations in housekeeping genes related to AMR without the need to state each isolate’s species beforehand. Also, the optional mash analysis for the clusterization of the analyzed genomes does not require the input of a reference genome. Quickness and reliability have also guided our choice regarding which tools to include in the workflow. Most of the software we selected has been already in use for many years, representing some of the most trusted tools in the microbiology community. An example like SPAdes [22], which was developed more than 10 years ago, is regarded today as a de facto standard for a bacterial genome’s assembly, especially when working with short-read sequencing data. This success is likely attributable to the optimization of its innovative algorithms for prokaryotic genomes, which over time led to its broad adoption among microbiology professionals and integration in many modern pipelines and platforms (like Galaxy [23]). Along these lines, we chose to rely on these renowned tools with the intent of maximizing efficiency and reliability of results, developing a product which can handle large numbers of bacterial isolates.

Regarding the management of all tools and environments, Nextflow [7] proved to meet the high expectations set by the bioinformatic community in recent years. While still requiring a steep learning curve, this DSL did in fact simplify many of the most critical steps in developing an automated pipeline like Enteroflow. Testing and development were two of the stages which benefited the most from the self-tracking capabilities built in the management system (see pipeline metrics reports and resumability of interrupted workflows). Moreover, its processes’ structure intrinsically implies their parallelization between computing cores, allowing for a sensible reduction in overall processing times.

The assembly quality “test” indicated an optimum result as the genomes were nearly fully reconstructed, with low SNP variants (identity 99.9%). The QUAST output obtained, both for the quality test than for the performance test, indicated that the assembly follows the quality recommendations of the EURL regarding contig number and divergence in total length [24].

Future releases of Enteroflow will not be limited to the correction of possible emerging bugs and errors, as it is already our intention to implement new features for the analysis of other multi-drug resistant (MDR) bacterial species of public health concern, also included in EU decision 1729/2020 [2]. For this purpose, we are planning to include a tool for species identification, which will also allow the use of species-specific point mutations databases.

In conclusion, Enteroflow noticeably reduces the processing times of WGS workflows for the bioinformatic analyses of *Enterococcus* spp. isolates, including typically used databases for AMR monitoring, while also providing a harmonized frame for consultation and comparison of results that is flexible and may be improved according to future laboratory needs.

## 4. Materials and Methods

### 4.1. Installation and Dependencies

Before Enteroflow’s execution, the following tools must be pre-installed in the Operating System: Conda v25.1.1 (or previous) [15] and the latest releases of Nextflow [7], Blast [16] and KMA [17]. Should the user intend to deploy the Kraken2 [18] option to obtain a taxonomical classification of the isolates, the installation of this tool and its database (circa 90 GB) is mandatory before Enteroflow’s deployment. Results will then be graphically represented via Mashtree [19], which, by contrast, is installed autonomously via Conda [15].

**Figure 5:**
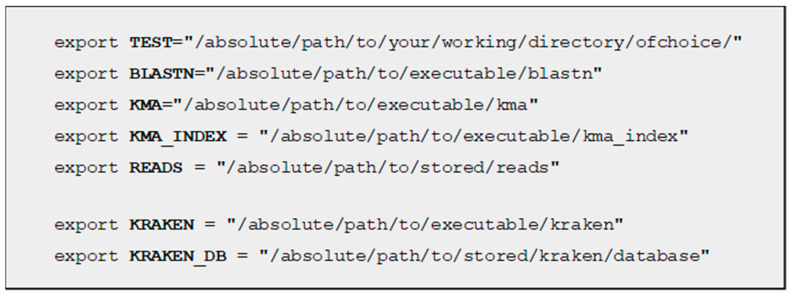
Environment variable definitions for a Unix/Linux shell.

Enteroflow’s execution relies on 5 mandatory environment variables (plus 2 optional ones), which must be set by the user when starting a new bash session or permanently added to the .bashrc file in the $HOME directory for all future bash sessions (Figure 5). They contain the absolute positions of such tools inside the OS, allowing Enteroflow’s execution, regardless of how the file system has been previously organized.

### 4.2. Pipeline Architecture

The workflow is structured in 4 main phases: (i) quality control of input raw reads, (ii) de novo assembly, (iii) genotyping and molecular characterization, and (iv) presentation of summarized results.

i. Quality control

Quality control steps begin with paired-end raw sequencing reads produced by any Illumina sequencing device, which undergo adapters’ removal and filtering by quality (>Q25 for a minimum of 60% of bases in a single read) with the FastP(v1.0.1) tool from OpenGene [25]. These same reads are passed to the FastQC(v0.12.1) tool by Babraham Bioinformatics for quality statistics (pre and post filtering) [26] and to the MultiQC(v1.31) tool from Seqera [27] to combine all results into a single .html report file. This phase allows the correct manipulation of raw reads, ensuring necessary minimum read quality and reliability for the following stages of the workflow while allowing the user to evaluate the output from the sequencing run.

ii. De Novo assembly

Filtered raw reads then proceed to the de novo assembly steps operated by SPAdes (St. Petersburg Genome Assembler, [22]), a genome assembly toolkit which uses a multi-sized de Bruijn graph approach. Its iterative method drastically improves the assembly accuracy of small genomes, with the resolution of repeated sequences and the correction of sequencing errors, when compared with single k-mer strategies.

iii. Genotyping and molecular characterization

Resulting assemblies are subsequently passed to the genotyping and characterization steps which include the research of AMR-related genes and point mutations, virulence genes, known replicon plasmidic sequences and MLST. A mass screening of the assembled contigs for AMR genes and point mutations is obtained via ABRicate (v1.0.1) [28] in liaison with ResfinderDB (v2.6.0) [29] and PointfinderDB (v4.1.1) [30] and with AMRfinder (v4.0.3) [31] through its own database, with a minimum coverage of 60% and a minimum identity of 90%. The use of multiple tools and different databases not only allowed us to include AMR-related point mutations in our results, but first and foremost increased the reliability of the detected AMR genes by comparing different sources. The same tool is employed for the detection of virulence-related genes and of known plasmid sequences using two more specifically curated databases (VirulencefinderDB (v2.0.1) [32] and PlasmidfinderDB (v2.2.0) [33], respectively). The MLST tool (v.2.23.0) [34] has been introduced, together with PubMLST ‘s database [20,21,35] to confirm the identification of the *Enterococcus* species, also providing a Sequence Type (ST) when available. Other than for comparison purposes, such genomic information may prove to be useful during the final interpretation of results, where certain AMR profiles may be common in certain species/STs, while raising attention in other ones.

All databases can be updated via an automated script inside Enteroflow’s code, simply by setting the --update_db parameter to “yes”(default=”no”) when running the pipeline’s main command. This option becomes obviously mandatory when running Enteroflow for the first time on a new device as the databases are required to be downloaded rather than just updated.

iv. Presentation of summarized results.

Results produced by the genotyping and molecular characterization phase converge in a custom R script, which is designed to process multiple .txt files from a specified input directory and compile them into a single Excel workbook by using the *openxlsx* package for Excel file creation and manipulation. Upon execution, the script dynamically imports all resulting .txt files, filters out rows containing specific keywords, and re-writes their content into individual Excel sheets named after each input file. The final workbook is saved into the data/annotation folder in the user-defined run path, together with all results generated by individual processes. All .html reports generated from the quality control phase are stored inside the data/fastqc and data/multiqc folders, while trimmed reads and .fasta assemblies can be found in the data/fastp and data/spades folders, respectively.

Enteroflow’s source code is publicly available as a github repository: https://github.com/CRAB-IZSLT/EnteroFlow.

### 4.3. Quality Testing

Quality testing was performed on publicly available short raw reads (SRR6768163, SRR6768428, SRR6768327, SRR6768232, SRR6768236) and their corresponding long read assembly from the same isolates and authors were used as references [36]. The five assemblies obtained with Enteroflow were mapped against their own references using minimap2 [37]. Parameters for evaluation of the quality showed deviations in size always less than 10% of the reference genome size, the number of resulting contigs lower than 200, and very high identity with the reference sequence (97–99%) [36].

### 4.4. Benchmarking

In total, 10 random *Enterococcus* spp. isolates from our collection (PRJEB96944) were analyzed in parallel using Enteroflow, Nullarbor [10], and Bactopia [11], all on default settings with a 16-core CPU and 64 GB of RAM available locally. Comparison focused on both qualitative parameters, like type of results and requirements, and quantitative ones like duration time and RAM usage. The final outputs related to MLST, AMR genes, and plasmid content generated by each pipeline were compiled to enable a rigorous comparison.

## Figures and Tables

**Table 1:** Statistics of Enteroflow’s short read assemblies and their alignment to reference sequences, using Minimap2.

		SRR6768163	SRR6768232	SRR6768236	SRR6768327	SRR6768428
**Assembly**	**N° contigs (>1000 bp)**	183	183	157	165	179
	**Total length**	2,973,147 bp	3,019,906 bp	2,966,970 bp	2,947,552 bp	2,911,576 bp
	**Largest contig**	180,945 bp	158,984 bp	124,974 bp	117,522 bp	100,544 bp
	**N50**	34,377 bp	38,672 bp	45,124 bp	39,545 bp	36,946 bp
	**N90**	6992 bp	7473 bp	10,553 bp	8733 bp	7339 bp
	**auN**	49,162.4	51,633.3	49,986.1	47,108.9	41,424.1
	**L50**	23	22	21	23	25
	**L90**	95	90	78	79	92
	**N° N’s per 100 kps**	24.39	26.34	13.74	17.53	17.06
**Alignment to ** **Reference**	**N° mapped contigs**	167	152	140	141	198
	**Identical sites**	2,715,830	2,710,104	2,721,660	2,597,602	2,724,329
	**Identity**	99.9%	99.9%	99.9%	99.9%	99.8%
	**Ref. chromosome length**	2,883,877 bp	2,855,729 bp	2,863,087 bp	2,731,844 bp	2,912,017 bp
	**Coverage of Ref.**	94.2% (2,717,947)	95.0% (2,711,659 bp)	95.1% (2,723,740 bp)	95.1% (2,599,315 bp)	93.7% (2,728,020 bp)

**Table 2:** Summarization of the differences in RAM usage and execution times between the three compared pipelines (N.A.: Not available).

	Execution Time	Max Ram	Output File
**Enteroflow**	52 min 55 s	9048 GB	One Excel + txt file
**Nullarbor**	2 h 1 min 57 s	N.A.	Several tsv and txt files
**Bactopia**	2 h 26 min 36 s (sum of 10 runs)	8039 GB (mean of 10 runs)	Subdirectories

## Data Availability

Enteroflow’s source code is publicly available as a github repository: https://github.com/CRAB-IZSLT/EnteroFlow.

## Supplementary Materials

The following supporting information can be downloaded at https://www.mdpi.com/article/10.3390/ijms26199441/s1.

## Abbreviations

The following abbreviations are used in this manuscript (order of appearance):

AMR	Antimicrobial Resistance
CRN-AR	National Reference Center for Antimicrobial Resistance
NRL-AR	National Reference Laboratory for Antimicrobial Resistance
IZSLT	Istituto Zooprofilattico Sperimentale del Lazio e della Toscana
E.	*Enterococcus*
HTS	High-Throughput Sequencing
DSL	Domani Specific Language
WHO	World Health Organization
EU	European Union
HGT	Horizontal Genetic Transfer
MGEs	Mobile Genetic Elements
VRE	Vancomycin-Resistant Enterococci
MLST	Multi-Locus Sequence Typing
MDR	Multi-drug Resistant
ST	Sequence Type
